# Combined CA125, NSE, and multiple inflammatory indices for diagnosis of oral squamous cell carcinoma

**DOI:** 10.3389/fonc.2025.1543055

**Published:** 2025-05-19

**Authors:** Kudelaiti Abudukelimu, Ailifeire Tuerxuntayi, Aikepaer Aierken, Rexiati Keranmu, Duolikun Wufuer

**Affiliations:** ^1^ Department of Oral and Maxillofacial Surgery, People’s Hospital of Xinjiang Uygur Autonomous Region, Urumqi, China; ^2^ Department of Gastroenterology, People’s Hospital of Xinjiang Uygur Autonomous Region, Urumqi, China; ^3^ Department of Medical Research and Translational Management, People’s Hospital of Xinjiang Uygur Autonomous Region, Urumqi, China

**Keywords:** neutrophil-to-lymphocyte ratio, platelet-to-lymphocyte ratio, systemic inflammation response index, carbohydrate antigen 125, neuron-specific enolase, oral squamous cell carcinoma, diagnostic value

## Abstract

**Objective:**

To investigate the changes of serum carbohydrate antigen 125 (CA125), neuron-specific enolase (NSE), neutrophil-lymphocyte ratio (NLR), platelet-lymphocyte ratio (PLR), and systemic inflammation response index (SIRI) in patients with oral squamous cell carcinoma(OSCC) and their diagnostic value for OSCC.

**Subjects and methods:**

A retrospective analysis was conducted on 136 patients with oral squamous cell carcinoma (OSCC) and 34 healthy controls. Blood routine parameters, as well as serum levels of CA125 and NSE, were obtained for the patients. Additionally, the neutrophil-to-lymphocyte ratio (NLR), platelet-to-lymphocyte ratio (PLR), and systemic inflammation response index (SIRI) were calculated. The diagnostic value of each marker, both individually and in combination, was evaluated using receiver operating characteristic (ROC) curve analysis.

**Results:**

We found that the levels of CA125, NSE, NLR, PLR, and SIRI in the oral squamous cell carcinoma group were significantly higher than those in the healthy control group (*P*_CA125_<0.001, *P*_NSE_=0.004, *P*_NLR_=0.034, *P*_PLR_=0.035, *P*_SIRI_=0.012). Significant differences were observed in NLR, PLR, and SIRI with respect to the size of the primary tumor and local lymph node involvement, as well as substantial differences in NLR and SIRI with distant metastasis (*P*<0.05). When NLR, PLR, and SIRI were combined with CA125 and NSE, the area under the curve (AUC) significantly increased (*P*<0.05). Further analysis using Delong’s test revealed a statistically significant difference in AUC values, suggesting that the combined diagnostic approach was more effective than individual markers.

**Conclusion:**

The diagnostic efficiency of OSCC can be enhanced by combining CA125, NSE, NLR, PLR, and SIRI. This combined approach offers high sensitivity and specificity for early diagnosis.

## Introduction

Oral squamous cell carcinoma (OSCC) is the most common malignant tumor of the oral cavity, with a 5-year survival rate of only 50-60%, resulting in approximately 50,000 deaths annually. It has become an increasingly significant global public health issue (1). Current management of OSCC relies primarily on surgical resection, complemented by radiotherapy and chemotherapy, highlighting the crucial role of comprehensive treatment in improving patients’ prognosis. However, despite advancements in surgical techniques and adjuvant therapies, the 5-year survival rate for patients with advanced or recurrent tumors has not improved significantly in recent years (2). Therefore, identifying reliable cancer markers for early intervention in OSCC is critical. Numerous studies have shown that inflammatory responses and nutritional status are key factors in tumor development (3). Non-specific indicators such as the platelet-to-lymphocyte ratio (PLR), neutrophil-to-lymphocyte ratio (NLR), and systemic inflammation response index (SIRI) are closely linked to tumor progression and prognosis (4). Serum tumor markers are simple, easily detectable, and widely used in clinical tumor screening (5). Carbohydrate antigen 125 (CA125) is typically produced by the embryonic digestive tract epithelium, amniotic membrane, adult pleura, and peritoneal mesothelial cells (6). However, its abnormal overexpression has been observed in various malignant tumors, including ovarian, pancreatic, bladder, lung, and OSCC. CA125 exhibits high specificity and sensitivity for early-stage OSCC diagnosis (7), making it a potential diagnostic marker for OSCC. Neuron-specific enolase (NSE), a nerve- and neuroendocrine-specific isozyme of enolase, plays a crucial role in aerobic glycolysis. As a standard serum tumor marker, NSE shows elevated expression in small-cell lung, prostate, and thyroid cancers (8–10).

In this study, we investigated the changes in serum CA125, NSE, and various inflammatory indexes in patients with oral squamous cell carcinoma and evaluated their diagnostic value for OSCC. The findings aim to offer valuable insights into the clinical diagnosis of OSCC.

## Materials and methods

### Participants

We retrospectively analyzed a total of 136 patients with oral squamous cell carcinoma (OSCC) who were admitted to the Department of Maxillofacial Surgery at the Xinjiang Uygur Autonomous Region People’s Hospital from January 2018 to December 2022, forming the OSCC group. Additionally, 34 healthy subjects admitted during the same period were selected as the control group. Inclusion criteria for the OSCC group were: (1) all patients underwent radical surgical resection and were diagnosed with OSCC based on postoperative pathology; (2) blood routine indices, as well as CA125 and NSE levels, were collected within one week prior to surgery. Exclusion criteria included incomplete medical history or blood test data, preoperative radiotherapy, the presence of other concurrent tumors, and lack of an obvious etiology for infection. Tumor staging was performed according to the seventh edition of the AJCC/TNM classification (11). The OSCC group consisted of 72 males and 64 females, aged 20–85 years, with a mean age of 57.99 ± 12.34 years. The healthy control group consisted of 19 males and 15 females, aged 20–80 years, with a mean age of 51.15 ± 12.37 years. There were no significant differences in gender or age between the two groups. Healthy controls (n=34) were selected from individuals undergoing routine health check-ups at our hospital during the same period. Inclusion criteria for controls were: (1) no history of malignancy, autoimmune diseases, or acute/chronic infections; (2) normal results on blood tests and imaging examinations; (3) age and sex matched to the OSCC group as closely as possible.

### Methods

Each patient provided a 2 mL fasting venous blood sample, collected in both an EDTA-K2 anticoagulant tube and a dry tube. The total blood cell count was determined using a Beckman 780 (Beckman Coulter, Brea, USA). The absolute values of neutrophils, lymphocytes, monocytes, and platelets were calculated from these results. Based on these values, the following parameters were derived: neutrophil-to-lymphocyte ratio (NLR) [NLR = neutrophils (×10^9^/L)/lymphocytes (×10^9^/L)], platelet-to-lymphocyte ratio (PLR) [PLR = platelets (×10^9^/L)/lymphocytes (×10^9^/L)] (12), and systemic inflammation response index (SIRI) [SIRI = neutrophils (×10^9^/L) × monocytes (×10^9^/L)/lymphocytes (×10^9^/L)] (13). CA125 levels were measured using the ARCHITECT i2000SR (Abbott Diagnostics, Abbott Park, USA), while neuron-specific enolase (NSE) levels were detected using the Roche E602 (Roche Diagnostics GmbH, Mannheim, Germany). The Ethics Committee of the People’s Hospital of Xinjiang Uygur Autonomous Region approved the collection of clinical and laboratory data, in accordance with the principles outlined in the Declaration of Helsinki.

### Statistical analysis

The data were analyzed using R version 4.2.3 (R Foundation for Statistical Computing, Vienna, Austria), SPSS 26.0 (IBM Corp., Armonk, NY, USA), and GraphPad Prism 8 (GraphPad Software, San Diego, CA, USA). For normally distributed data, the mean ± standard deviation ( ± s) was used to express the values, and the t-test was applied for between-group comparisons. Non-normally distributed data were expressed as the median (interquartile range), and the Mann-Whitney U test was used for between-group comparisons. Categorical data were presented as counts (%), and comparisons between groups were performed using the χ² test. Receiver operating characteristic (ROC) curve analysis was conducted to determine the cut-off values, sensitivity, specificity, and area under the ROC curve (AUC) of CA125, NSE, NLR, PLR, and SIRI, both individually and in combination, to assess their diagnostic efficacy for OSCC. Delong’s test was applied to compare differences in ROC curves. R packages, such as rROC, pROC, and pheatmap, were used to generate ROC curves for evaluating combined diagnostic efficacy. A p-value of *< 0.05* was considered statistically significant. Optimal cut-off values for NLR, PLR, SIRI, CA125, and NSE were determined using the Youden Index (*J = sensitivity +* sp*ecificity − 1)* to maximize diagnostic accuracy. Bootstrapping (1000 resamples) was applied to address potential biases arising from sample size imbalance.

## Results

### Patient characteristics

**Figure 1:**
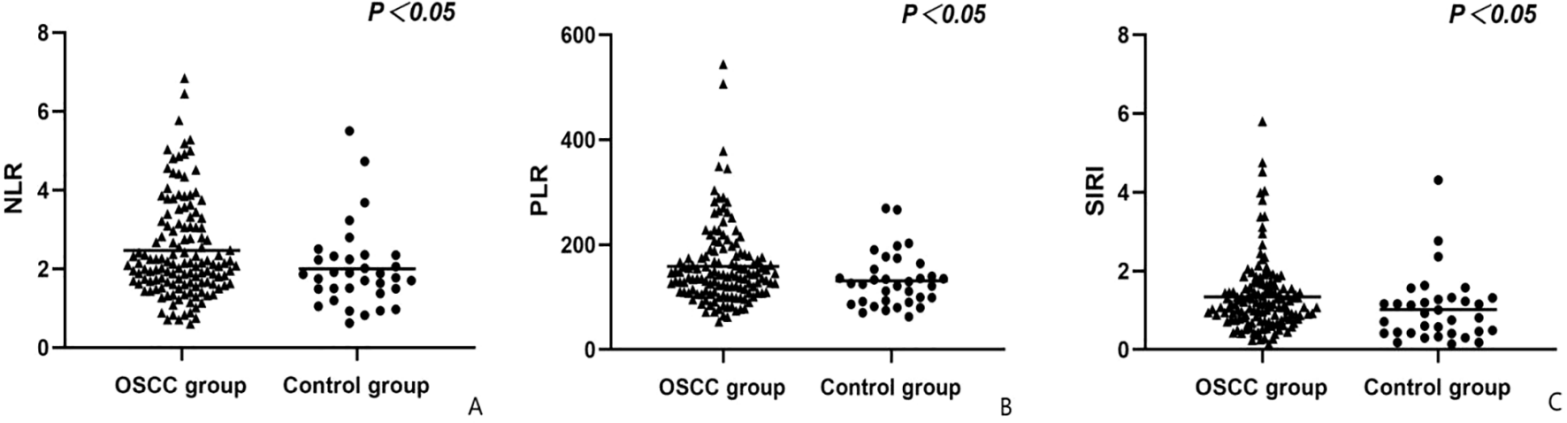
Comparison of groups in terms of NLR **(A)**, RDW **(B)** and SIRI **(C)** values. NLR, Neutrophil-lymphocyte ratio; PLR, Platelet-lymphocyte ratio; SIRI, Systemic inflammation response index.

Our study included two groups (**Table 1**): the OSCC group, which comprised 136 participants (72 males, 64 females; ages 20 to 85, mean age 57.99 ± 12.34 years), and the healthy control group, which comprised 34 participants (19 males, 15 females; ages 20 to 80, mean age 51.15 ± 12.37 years). There were no significant differences in gender, WBC, neutrophil, or platelet levels between the two groups (*P>0.05*). Although the OSCC group had a slightly higher mean age (57.99 vs. 51.15 years, *P=0.002*), age-adjusted logistic regression confirmed that biomarker differences remained statistically significant (*P<0.05* for all). CA125 and NSE levels were significantly higher in the OSCC group compared to the healthy control group (*P*_CA125_<0.001, *P*_NSE_=0.004), while significant differences in NLR, PLR, and SIRI values were observed between the two groups (*P*_NLR_=0.034, *P*_PLR_=0.035, *P*_SIRI_=0.012; **Figure 1**).

**Table 1:** Comparison of clinical features and laboratory indicators among two groups.

Variable	OSCC group	Healthy control group	*P*
n	136	34	
Age (years)	57.99 ± 12.34	51.15 ± 12.37	0.002
Sex (%)			0.976
male	72 (52.94%)	19 (55.88%)	
female	64 (47.06%)	15 (44.12%)	
WBC (10^9^/L)	6.185 (4.915-7.095)	6.815 (4.895-7.955)	0.223
Neutrophil (10^9^/L)	3.685 (2.805-4.435)	3.850 (2.303-4.820)	0.687
Lymphocyte (10^9^/L)	1.695 (1.225-2.133)	2.045 (1.588-2.390)	0.005
Monocyte (10^9^/L)	0.500 (0.410-0.640)	0.430 (0.308-0.580)	0.028
Platelet (10^9^/L)	234.5 (191.5-280.8)	266.5 (204.5-308.5)	0.088
PLR	141.7 (109.6-179.0)	125.4 (91.4-156.2)	0.035
NLR	2.124 (1.622-3.092)	1.821 (1.461-2.329)	0.034
SIRI	1.153 (0.780-1.558)	0.864 (0.424-1.287)	0.012
CA 125 (ng/ml)	20.75 (12.53-28.28)	11.90 (8.47-15.33)	<0.001
NSE (ng/ml)	15.06 (12.11-17.94)	13.59 (9.56-15.12)	0.004

### Associations between NLR, PLR,SIRI,CA 125,NSE and clinicopathological characteristics of 136 OSCC patients

According to the findings presented in **Table 2**, NLR, PLR, and SIRI showed significant correlations with primary tumor size and local lymph node involvement (*P*<0.05). Additionally, NLR and SIRI exhibited strong associations with distant metastasis (*P*<0.05). However, CA125 and NSE did not show significant correlations with primary tumor size, local lymph node involvement, or distant metastasis in oral squamous cell carcinoma (*P*>0.05).

**Table 2:** Correlations between clinicopathological features and pre-operative NLR, PLR, SIRI, CA125 and NSE in Oral squamous cell carcinoma.

Variable	N	NLR	PLR	SIRI	CA125	NSE
The size of the primary tumor (T stage)						
T1+T2	75	1.908 (1.525-2.414)	134.3 (106.1-161.1)	1.016 (0.657-1.349)	22.40(12.80-28.40)	14.95 (11.99-18.10)
T3+T4	61	2.477 (1.861-3.868)	149.2 (120.3-212.9)	1.392 (0.909-1.910)	18.40(10.85-27.40)	15.27 (12.29-17.92)
*P*		0.001	0.017	0.005	0.423	0.885
Lymph node metastasis (N stage)						
N0-N1	114	1.968 (1.570-2.749)	134.7 (107.6-167.1)	1.039 (0.729-1.412)	20.75(12.08-28.13)	15.16 (12.04-17.98)
N2-N3	22	3.768 (2.228-4.899)	180.2 (142.6-236.8)	1.793 (1.422-3.851)	19.65(14.93-28.58)	14.09 (12.19-17.40)
*P*		<0.0001	0.001	<0.0001	0.587	0.618
Distant metastasis (M stage)						
M0	128	2.089 (1.617-3.031)	138.2 (108.8-177.6)	1.089 (0.776-1.507)	21.00(12.60-28.40)	15.17 (12.28-17.93)
M1	8	4.685 (2.629-2.629)	165.5 (122.9-222.1)	3.592 (1.265-4.401)	18.15(11.13-24.70)	13.24 (8.04-20.81)
*P*		0.008	0.225	0.006	0.465	0.605

### Diagnostic value of NLR, PLR, SIRI, CA125 and NSE alone or in combination for oral squamous cell carcinoma

**Figure 2:**
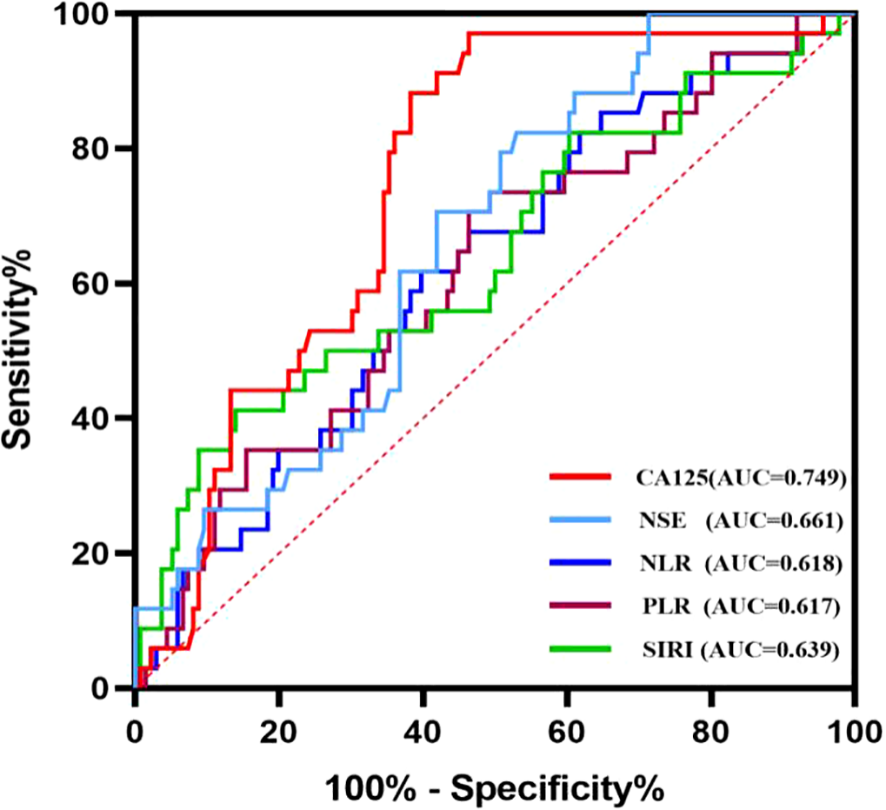
Diagnostic value of NLR, PLR, SIRI, CA125 and NSE alone for oral squamous cell carcinoma.

Based on the correlation between relevant indicators and oral squamous cell carcinoma (OSCC), a receiver operating characteristic (ROC) curve was constructed to determine the cut-off values, along with their corresponding sensitivity and specificity values (**Figure 2**). The results are presented in **Table 3**. When individual indicators were used for OSCC diagnosis, the area under the curve (AUC) values for neutrophil-to-lymphocyte ratio (NLR), platelet-to-lymphocyte ratio (PLR), systemic immune-inflammation index (SIRI), cancer antigen 125 (CA125), and neuron-specific enolase (NSE) were 0.618, 0.617, 0.639, 0.749, and 0.661, respectively. The corresponding sensitivities were as follows: NLR—60.3%, PLR—53.7%, SIRI—86.0%, CA125—53.7%, and NSE—47.1%. The specificities were observed as follows: NLR—61.8%, PLR—70.6%, SIRI—41.2%, CA125—97.1%, and NSE—82.4%. Overall, each biomarker demonstrated promising diagnostic capabilities for OSCC detection; however, SIRI exhibited higher sensitivity, while CA125 and NSE showed superior specificity.

**Table 3:** Diagnostic efficiency of NLR, PLR, CEA used alone and their combined use for oral squamous cell carcinoma.

Variable	AUC (95%CI)	*P-*value	Sensitivity/%	Specificity/%	Cut-off
NLR	0.618 (0.515-0.721)	<0.0001	60.3	61.8	1.928
PLR	0.617 (0.569-0.754)	0.001	53.7	70.6	136.35
SIRI	0.639 (0.528-0.751)	0.014	86.0	41.2	0.606
CA125	0.749 (0.668-0.830)	<0.0001	53.7	97.1	18.95
NSE	0.661 (0.569-0.754)	0.001	47.1	82.4	15.535
NLR+CA125	0.781 (0.704-0.858)	<0.0001	66.9	88.2	0.187
PLR+CA125	0.790 (0.709-0.871)	<0.0001	75.0	79.4	0.242
SIRI+CA125	0.772 (0.693-0.850)	<0.0001	66.2	85.3	0.194
NLR+NSE	0.715 (0.624-0.807)	<0.0001	49.3	88.2	0.143
PLR+NSE	0.705 (0.608-0.802)	<0.0001	55.1	82.4	0.165
SIRI+NSE	0.706 (0.611-0.802)	<0.0001	64.7	70.6	0.200
NLR+CA125+NSE	0.841 (0.770-0.913)	<0.0001	80.1	79.4	0.239
PLR+CA125+NSE	0.835 (0.756-0.914)	<0.0001	77.2	82.4	0.233
SIRI+CA125+NSE	0.835 (0.761-0.908)	<0.0001	65.4	94.1	0.157
NLR+PLR+SIRI+CA125+NSE	0.843 (0.770-0.916)	<0.0001	77.9	82.4	0.229

* Cut-off values were derived using the Youden Index (J = sensitivity + specificity − 1)

**Figure 3:**
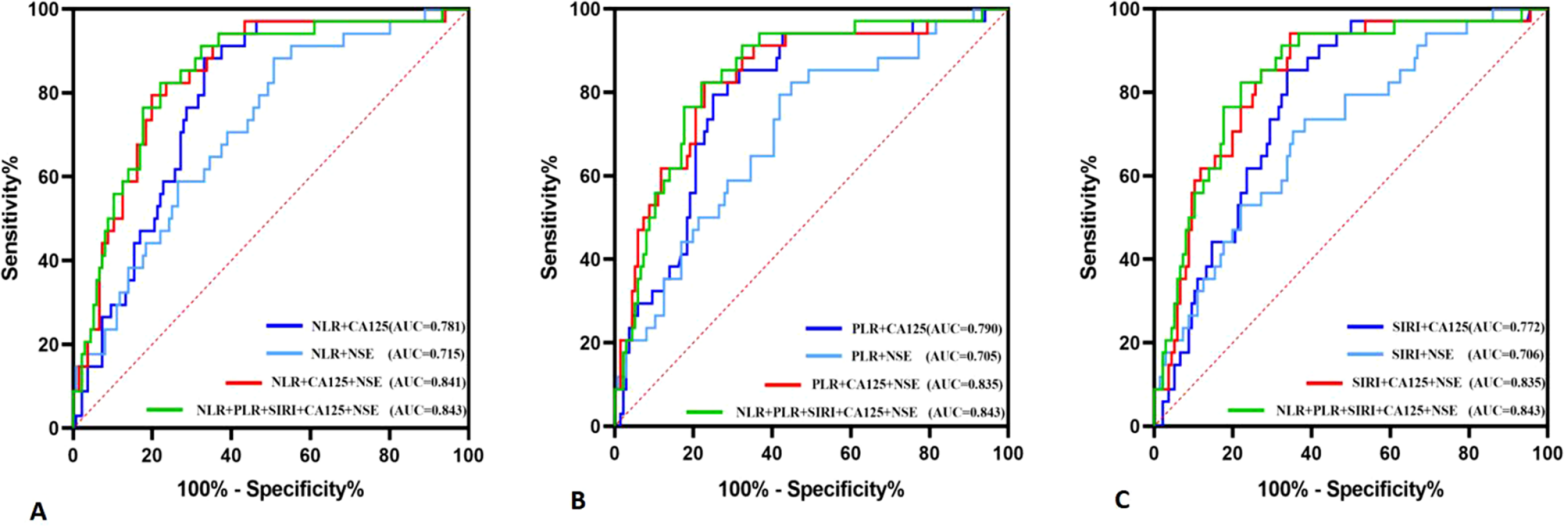
Diagnostic value of NLR, PLR, and SIRI combined with CA125 and NSE diagnosis in oral squamous cell carcinoma. **(A)** NLR co-diagnosis **(B)** PLR co-diagnosis **(C)** SIRI co-diagnosis.

**Figure 4:**
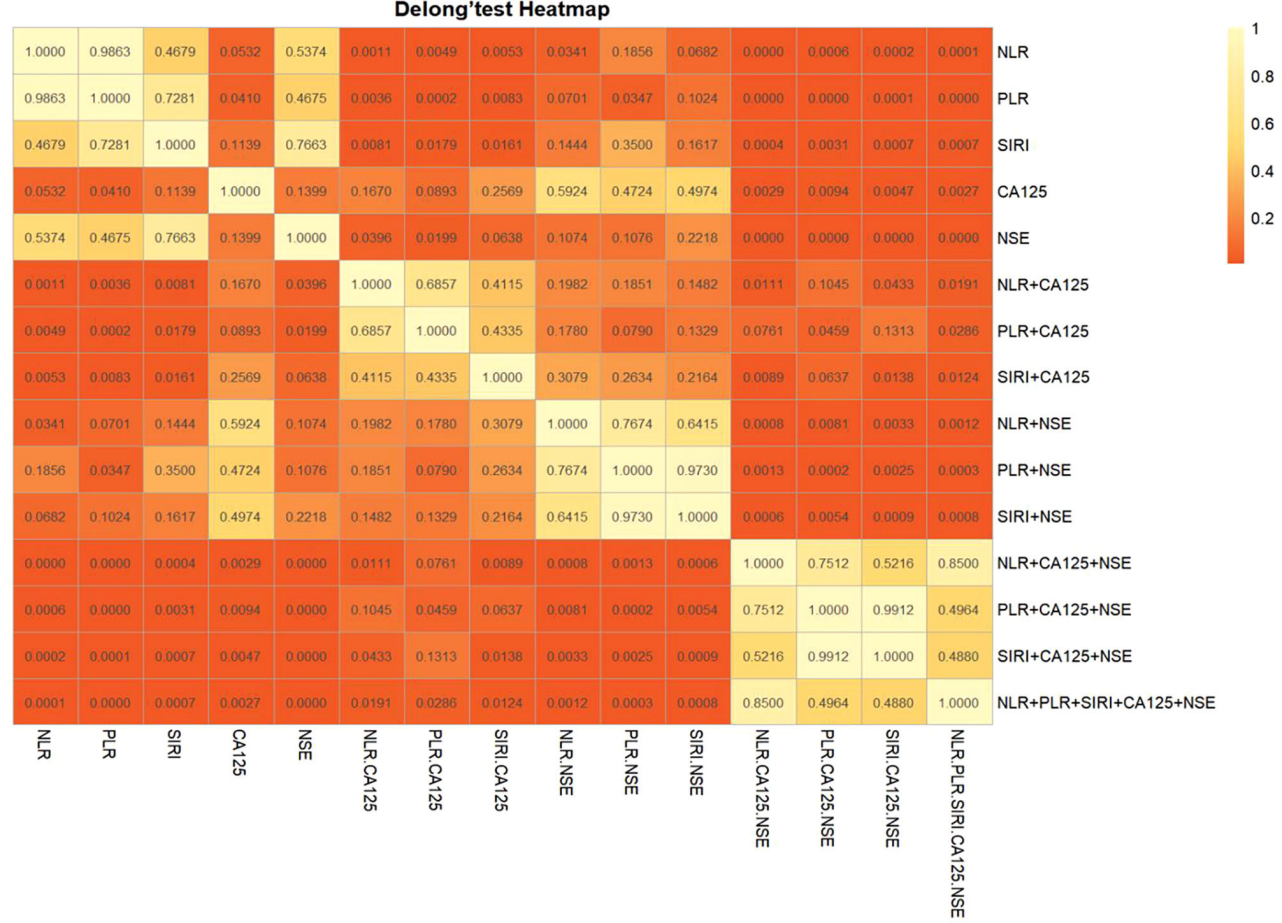
Results of Delong’ test between indicators.

The diagnostic system combining CA125, NSE, NLR, PLR, and/or SIRI was evaluated using a multivariate logistic regression model, as shown in **Table 3**. **Figure 3A** presents the ROC curve for diagnosing OSCC with NLR combined with CA125 and/or NSE. The area under the curve (AUC), sensitivity, and specificity for NLR combined with CA125 were 0.781, 66.9%, and 88.2%, respectively. When NLR was combined with NSE, the AUC was 0.715, with sensitivity and specificity values of 49.3% and 88.2%, respectively. When all three indicators were combined, the AUC increased to 0.841, with sensitivity remaining at a high level of 80.1% and specificity reaching 79.2%. **Figure 3B** illustrates the ROC curve for PLR combined with CA125 and/or NSE in OSCC diagnosis. The AUC, sensitivity, and specificity for PLR combined with CA125 were 0.790, 75.0%, and 79.5%, respectively, suggesting its potential as a diagnostic tool for OSCC. Similarly, when PLR was combined with NSE, the AUC, sensitivity, and specificity values were 0.705, 55.1%, and 82.4%, respectively, indicating its utility in diagnosing OSCC. Combining all three indicators improved diagnostic performance, with an AUC of 0.835, sensitivity of 77.2%, and specificity of 82.4%. **Figure 3C** shows the ROC curve for SIRI combined with CA125 and/or NSE in OSCC diagnosis. The AUC, sensitivity, and specificity for the combination of SIRI and CA125 were 0.772, 66.2%, and 85.3%, respectively. In contrast, for the combination of SIRI and NSE, the AUC, sensitivity, and specificity were 0.706, 64.7%, and 70.6%, respectively. When all three indicators were combined, the AUC improved to 0.835, with sensitivity of 65.4% and specificity of 94.1%. The combined diagnostic model using CA125, NSE, NLR, PLR, and SIRI yielded an AUC of 0.843, with sensitivity of 77.9% and specificity of 82.4%. Overall, the integration of these five indicators compensates for the limited diagnostic efficiency observed with individual markers, while ensuring high sensitivity and specificity. Furthermore, Delong’s test confirmed that the ROC of the combined model significantly outperformed the individual biomarkers (**Figure 4**). Based on the differences in AUC, sensitivity, and specificity observed in the ROC analysis, it can be concluded that the combined application of CA125, NSE, NLR, PLR, and SIRI provides superior diagnostic value compared to any single indicator.

## Discussion

Oral squamous cell carcinoma is a common malignancy in the head and neck region, with patients diagnosed with advanced OSCC typically surviving less than 30 months following complete surgical resection (14). Additionally, factors such as tumor location, progression, age, and underlying health conditions significantly influence the 5-year survival rate of affected individuals. As such, identifying reliable tumor markers for molecular diagnosis, prognostic prediction, and targeted therapy is essential for the effective management of OSCC.

Previous studies have highlighted the critical role of cancer-related inflammation in carcinogenesis and tumor progression (15). The underlying mechanisms involve complex interactions between inflammation, malnutrition, immune dysfunction, platelet activation, angiogenesis, and cytokine signaling (16). Both endogenous and exogenous inflammatory responses can induce immunosuppression, thereby increasing susceptibility to cancer. Additionally, these responses can promote malignant transformation by recruiting and activating inflammatory cells that provide support to cancer cells. Systemic inflammation further enhances tumor invasion and progression by inhibiting apoptosis and promoting angiogenesis (17). Hematological indicators, such as the neutrophil-to-lymphocyte ratio (NLR), platelet-to-lymphocyte ratio (PLR), and other blood parameters, are closely linked to host inflammation and nutritional status in cancer patients. These indicators have the potential to predict cancer progression and survival to some extent (18–20). Unlike traditional inflammatory markers, NLR, PLR, and the systemic immune-inflammatory response index (SIRI) are derived from the quantification of neutrophils, monocytes, and lymphocytes in the blood, offering a more comprehensive reflection of the body’s inflammatory and immune status. Moreover, they have the advantages of being simple to measure and cost-effective. Tumor markers like CA125 and NSE, which are produced by tumors, play a crucial role in assessing tumor occurrence, progression, recurrence, and metastasis (21). In this study, we retrospectively analyzed the values of NLR, PLR, SIRI, CA125, NSE, and related clinicopathological data in patients with OSCC to evaluate their potential as diagnostic tools.

The systemic inflammatory indices, including the neutrophil-to-lymphocyte ratio (NLR), platelet-to-lymphocyte ratio (PLR), and systemic immune-inflammatory response index (SIRI), are derived from the counts of neutrophils, lymphocytes, and platelets. Increasing evidence has highlighted the impact of platelets, neutrophils, monocytes, and lymphocytes on tumor biology (22). Factors released by these cells—such as intravascular aggregates, vascular endothelial growth factor (VEGF), transforming growth factor-beta (TGF-β), and platelet-derived growth factor (PDGF)—facilitate tumor cell differentiation, proliferation, and metastasis, while also contributing to tumor angiogenesis, invasion, and metastatic spread (23). Previous studies have identified neutrophils as the primary source of matrix metalloproteinase-9 (MMP-9) in precancerous tissues. By regulating VEGF to promote angiogenesis within tumors, MMP-9 plays a critical role in tumor invasion and metastasis (24). Furthermore, MMP-9 accelerates the proliferation of oncogene-expressing keratinocytes and aids their dissemination, promoting aberrant hyperplasia that can lead to malignant transformation while influencing the differentiation characteristics of newly formed tumors (25). Since CD4+ and CD8+ T cells are key in combating tumor cells, a reduced lymphocyte count often signifies an impaired tumor-specific immune response (26). In particular, the rapid progression of tumors is facilitated by the synergistic interaction between neutrophils and lymphocytes. Additionally, tumor cells activate platelets by expressing tissue factor (TF) on their membranes, triggering the plasma coagulation cascade and subsequent thrombin production (27). This process increases the expression of VEGF, PDGF, basic fibroblast growth factor (bFGF), and epidermal growth factor (EGF) stored in platelet α-granules, ultimately fostering tumor angiogenesis and accelerating tumor progression (28, 29). Moreover, VEGF enhances the intravascular metastasis of tumor cells, leading to the formation of distinct aggregates that significantly impact tumor metastasis (30). In essence, the inflammatory response regulates these blood components, promoting tumor initiation and progression. Consequently, monitoring alterations in NLR, PLR, and SIRI could be a valuable approach for early tumor diagnosis. The study by Hernandez-Ainsa (31) revealed a consistent increase in NLR and PLR from asymptomatic patients to those diagnosed with colorectal cancer. Additionally, a positive correlation was found between SIRI levels and the CD44+ tumor stem cell (CSC) score, with higher SIRI levels associated with an increased proportion of CD44+ CSCs (20). In our study, significantly elevated levels of NLR, PLR, and SIRI were observed in the OSCC group compared to the healthy control group. Moreover, significant differences in NLR, PLR, and SIRI were found in OSCC patients concerning primary tumor size, local lymph node involvement, and distant metastasis. These findings suggest that NLR, PLR, and SIRI could serve as potential markers for assessing disease progression in OSCC.

Tumor markers such as CA125 and NSE are well-established indicators of cancer, with CA125 elevated in various malignancies, including ovarian, breast, cervical, pancreatic, and colorectal cancers (32–34). CA125 interacts with specific ligands, including galectin-1/3, mesothelin, and Siglec-9, which play key roles in regulating tumorigenesis, progression, migration, invasion, and tumor immunity through various signaling pathways associated with cancer (35). On the other hand, NSE is expressed primarily in the cytoplasm of central and peripheral neurons, as well as neuroendocrine cells under normal physiological conditions (36). Elevated NSE levels, resulting from overexpression or increased serum concentration, are associated with malignant tissue proliferation, highlighting its potential in cancer diagnosis, treatment, and prognosis (37). In our study, the serum levels of both CA125 and NSE were significantly higher in OSCC patients compared to healthy controls, indicating a statistically significant difference. However, no significant differences were observed in the serum levels of these markers based on primary tumor size, lymph node involvement, or distant metastasis in OSCC patients. This suggests that while these tumor markers exhibit high specificity for cancer diagnosis, their sensitivity is relatively lower when assessing tumor size, lymph node involvement, and distant metastasis.

In our study, we evaluated the diagnostic potential of combining serum tumor markers CA125 and NSE with inflammatory indices NLR, PLR, and SIRI for OSCC. The results revealed that several combinations of these markers significantly improved diagnostic accuracy, with the area under the receiver operating characteristic curve (AUC) exceeding 0.800 for multiple combinations. Notably, the combination of CA125, NSE, NLR, PLR, and SIRI yielded an AUC of 0.843, indicating high diagnostic efficacy. This performance is comparable to or surpasses that of other diagnostic panels reported in the literature. For example, a study on salivary biomarkers for OSCC diagnosis reported an AUC of 0.82 for a combination of microRNAs Salivary Biomarkers for Oral Squamous Cell Carcinoma Diagnosis: Current Status (38), while another study using serum protein combinations achieved an AUC of 0.78 Identification of potential salivary biomarker panels for oral squamous cell carcinoma (39). Our study’s diagnostic performance is particularly noteworthy given the non-invasive nature of blood-based tests, which are more accessible and practical for clinical application compared to saliva or tissue-based markers.

Comparing our findings with existing literature, while there are numerous studies on the prognostic value of NLR, PLR, and SIRI in OSCC, studies on their diagnostic value, especially in combination with tumor markers like CA125 and NSE, are limited. A study by Geng et al. (7) investigated the diagnostic value of saliva CA125 and tissue polypeptide specific antigen (TPS) in OSCC, finding elevated levels in patients with OSCC Saliva CA125 and TPS levels in patients with oral squamous cell carcinoma. However, our study focused on serum markers, which are more commonly used in clinical practice and offer greater convenience for routine screening. Another study by Wu et al. (40) evaluated serum levels of tumor markers in oral precancer patients and found significantly higher levels of carcinoembryonic antigen (CEA), squamous cell carcinoma antigen (SCC-Ag), and ferritin in these patients compared to healthy controls Serum levels and positive rates of tumor biomarkers in oral precancer patients. Although they did not specifically study CA125 or NSE, their findings support the use of serum tumor markers for early detection of oral malignancies.

The novelty of our study lies in the combination of inflammatory markers with traditional tumor markers for OSCC diagnosis. This approach leverages the systemic inflammatory response, which is known to play a significant role in cancer development and progression, with established tumor markers that reflect tumor burden. By integrating these different aspects of the disease, we achieved a more comprehensive diagnostic tool. This is particularly significant because inflammatory markers like NLR, PLR, and SIRI are simple, cost-effective, and widely available, making them ideal for use in resource-limited settings. Furthermore, the combination of these markers with CA125 and NSE provides a balanced assessment of both the host’s inflammatory response and the tumor’s biological characteristics, potentially enhancing diagnostic accuracy.

However, this study also has limitations. The retrospective design and sample size imbalance between the OSCC (n=136) and control groups (n=34) may introduce bias in specificity estimates. While bootstrapping was employed to mitigate this, external validation using independent cohorts is essential to confirm generalizability. Future prospective, multicenter studies with balanced cohorts are warranted to strengthen clinical applicability. Additionally, the lack of population-based validation underscores the need for broader demographic inclusion in subsequent research.

Our findings focused on the diagnostic utility of combined biomarkers in untreated OSCC patients. While the current data did not include post-surgical follow-up or relapse analysis, future studies should explore whether elevated NLR, PLR, SIRI, CA125, or NSE levels correlate with tumor relapse risk. Longitudinal monitoring of these biomarkers before and after surgery (e.g., 3–6 months post-operation) could clarify their dynamic changes and potential role in detecting residual disease or recurrence. Such analyses may further validate their prognostic value and guide personalized surveillance strategies.

## Conclusions

In conclusion, alterations in CA125, NSE, NLR, PLR, SIRI, and other markers—simple, cost-effective, and non-invasive biomarkers—may be closely associated with tumor onset and progression. The combined diagnostic value of CA125 and NSE, along with NLR, PLR, and SIRI, surpasses that of each individual marker. This advancement holds significant implications for the early detection of oral squamous cell carcinoma.

## Data Availability

The raw data supporting the conclusions of this article will be made available by the authors, without undue reservation.
